# TMJ pathomorphology in patients with JIA-radiographic parameters for early diagnosis-

**DOI:** 10.1186/s13005-018-0173-5

**Published:** 2018-09-17

**Authors:** Daniela Klenke, Anja Quast, Martina Prelog, Annette Holl-Wieden, Maximilian Riekert, Angelika Stellzig-Eisenhauer, Philipp Meyer-Marcotty

**Affiliations:** 1 Department of Orthodontics, University Medical Centre Göttingen, Poliklinik für Kieferorthopädie Robert-Koch-Str. 40 D-, 37075 Goettingen, Germany; 2 Paediatric Department, Paediatric Rheumatology, Würzburg University Hospital, Würzburg, Germany; 3 Department of Orthodontics, Würzburg University Hospital, Würzburg, Germany

**Keywords:** Juvenile idiopathic arthritis, TMJ, OPT, Asymmetry, Condyle pathomorphology

## Abstract

**Background:**

Juvenile idiopathic arthritis (JIA) is often accompanied by pathomorphological changes to the temporomandibular joint (TMJ). By analyzing orthodontical orthopantomograms of JIA patients the aims of the study were a) classification of condyle changes, b) quantification of bony asymmetries of condylar destruction and c) detection of relationships between disease duration and TMJ-involvement.

**Patients/Methods:**

46 caucasian JIA-patients (28 female; 18 male; < 16.0 years) were enrolled, each joint (*n* = 92) was morphologically assessed by means of orthopantomogram, quantitatively analysed and compared with duration of general disease. Condyle morphology was assessed using the Billiau scale for severity of destruction [1]. The quantitative analysis was based on ratios of condyle, ramus and mandible height.

**Results:**

Patients were divided into groups (Group I – slightly affected, *n* = 36; Billiau severity 0–2; condyle findings: X-ray normal, condyle erosions, condylar flattening; Group II – severely affected, *N* = 10; Billiau severity 3–4; condyle findings: condylar flattenings and erosions, unilateral/bilateral complete loss of condyles), based on morphological analysis of condylar destruction. Duration of disease was significantly longer in Group II (8.9 ± 5.2 years) than in Group I (4.6 ± 4.7 years). Asymmetries of condyle, ramus and mandible height, quantitatively analysed by contralateral comparison, were significantly more marked in patients of Group II than of Group I.

**Conclusions:**

Orthopantomogram imaging can be used in orthodontics clinical routine to detect TMJ-pathologies and is an important reference for monitoring progression of JIA. Classification into severe and slightly affected TMJ is possible by analysis of condylar pathomorphology. An association between degree of destruction, extent of lower jaw asymmetry and disease duration is suggested by the results.

## Background

Juvenile idiopathic arthritis (JIA) is a heterogeneous rheumatological disease of unknown cause, which occurs before the age of 16. The main clinical symptoms are a persistent swelling of one or more joints, restriction of joint mobility and pain on movement that has lasted for at least 6 weeks [2]. With a prevalence of 2.0 per 10,000 children, JIA is one of the most common chronic inflammatory-rheumatic diseases of childhood and adolescence [3]. Girls are affected about twice as often as boys [4].

The International League of Associations for Rheumatology classifies JIA into seven subgroups: systemic arthritis, oligoarthritis, rheumatoid factor -negative (RF-) polyarthritis, RF+ polyarthritis, psoriatic arthritis, enthesitis-associated arthritis and undifferentiated arthritis [2]. Oligoarthritis is the most frequent subtype of JIA, accounting for 27–56% of all cases. It often occurs in very early childhood and its peak onset is at two to 4 years of age [5].

Neither the aetiology nor the pathogenesis of JIA have been fully clarified to date [6]. The literature quotes frequencies between 17 and 87% for involvement of the TMJ in JIA [7–13].

Due to negative effects of the chronic inflammatory process, growth disorders of the TMJ and the entire craniofacial complex can become manifest even in childhood [14]. Tissue destruction in the TMJ causes the formation of granulation tissue, which replaces that destroyed and results in a pannus. This infiltrates and erodes the joint cartilage and adjacent bone, which eventually leads to condylar destruction or even complete loss of the condyle [14]. Subsequent characteristic growth disorders with underdevelopment of the mandibles, a posterior rotation of the lower jaw and skeletal class II with an anterior open bite have been described [15].

Involvement of the TMJ in JIA is often difficult to detect, because this can be asymptomatic [10, 16]. If symptoms occur, they often manifest as asymmetric mouth opening, joint noise, palpatory sensitivity of temporomandibular joints or associated muscles, rest pain in the temporomandibular joint, pain when chewing or limitation of jaw opening [17, 18]. Functional orthodontic therapy can reduce the discomfort of patients with JIA manifested in the jaw joint. Isola et al. were able to demonstrate a reduction of pain, click, crepitations and an improvement in the maximum oral opening after a 24-month therapy [19]. In extreme cases with the occurrence of ankylosis joint reconstruction is possible and leads to an postoperative improvement of mouth opening [20]. The temporomandibular joint involvement in JIA leads to a reduction in the quality of life [21, 22], which is adversely affected by pain and functional limitations [23].

If TMJ arthritis is symptomless, it is often only recognized by pediatricians and parents when there are already clear signs of growth disorders. As a result of delayed diagnostic, the therapy usually becomes very complex [24]. Overall, the identification of of temporomandibular joint arthritis in children with JIA is difficult since early signs or symptoms are absent in many patients [10, 16]. Diagnosis is therefore frequently delayed until pathomorphological changes with growth disorders are already obvious [25].

Typical pathomorphological changes of the TMJ present in JIA patients in the form of condylar flattening, osteophytes, erosions, sclerosis and subchondral cysts [18]. Asymmetry of the entire lower jaw has also been described [26, 27].

The panoramic radiograph or orthopantomogram (OPT) is a simple and rapidly usable method for demonstrating pathological changes to the TMJ in patients with JIA. The suitability of the OPT as a screening tool in JIA patients is currently under discussion.

With respect to abnormal condyle morphology a high specificity and sensitivity is described for the OPT when a TMJ synovitis is present [28, 29].

The aim of the study in patients with JIA was therefore to

- classify condylar pathomorphology of the TMJ on the basis of OPT,
- quantify asymmetry as a function of condylar destruction and
- analyse the relationship between disease duration and TMJ involvement in the same patients.

It is assumed that a classification of condylar pathomorphology and early signs of asymmetry is possible to detect in routine dental/ orthodontic imaging. Furthermore there could be an association between duration of disease and extent of destruction. Overall this could lead to an earlier diagnosis of JIA in the TMJ by recognizing TMJ involvement in the dental/ orthodontic routine OPG. Therefore this may improve the management of TMJ arthritis in JIA patients.

## Methods

### Patients

46 patients of Caucasian origin (28 female, 18 male) with an average age of 13.6 ± 3.5 years (range 7.4–19.1 years) were enrolled in the study. Inclusion criteria were diagnosis of JIA according to the criteria of the International League of Associations for Rheumatology including all seven subgroups of JIA.

Patients were acquired at the multidisciplinary outpatient rheumatology clinic of the Paediatric Department of Wuerzburg University Hospital, under the direction of an experienced paediatric rheumatologist (AHW). Exclusion criteria were congenital malformations, pre-existing traumas in the oral, maxillofacial and facial areas and malignancies. Inclusion criteria were diagnosed JIA according to the criteria of the International League of Associations for Rheumatology (ILAR), all seven subgroups of the JIA, age < 16.0 years at diagnosis, patients of Caucasian origin. Patients presenting a history of pain or difficulties at chewing or showing a restriction in mouth opening, deviations of the lower jaw or pain at palpitation of the TMJ in the clinical investigation were prospectively included into the study. Clinical functional analysis of the jaw was performed by an orthodontist (PMM).

### Methods

The study was conducted according to the institutional requirements of the Ethics Committee (File reference 42/13). All investigations were carried out under observance of the Declaration of Helsinki according to the principles of Good Clinical Practice.

TMJ pathomorphology was analysed on the basis of OPTs recorded during orthodontic treatment of the patient (X-ray machine: Orthophos DS®, Siemens, Erlangen, Germany). The software Sidexis XG (Version 2.56 of the company Sirona, Wals, Austria) was used to measure and assess the OPTs. The morphology of each joint (*n* = 96) was assessed individually and compared with the general duration of the disease. We defined general duration as period of time since diagnosis of JIA by a pediatric rheumatologist. In addition, quantitative assessments of mandibular morphology were undertaken to determine asymmetry indices.

All OPTs were analysed by two experienced examiners independently of each other.

### Morphological analysis of the TMJ

**Fig. 1:**
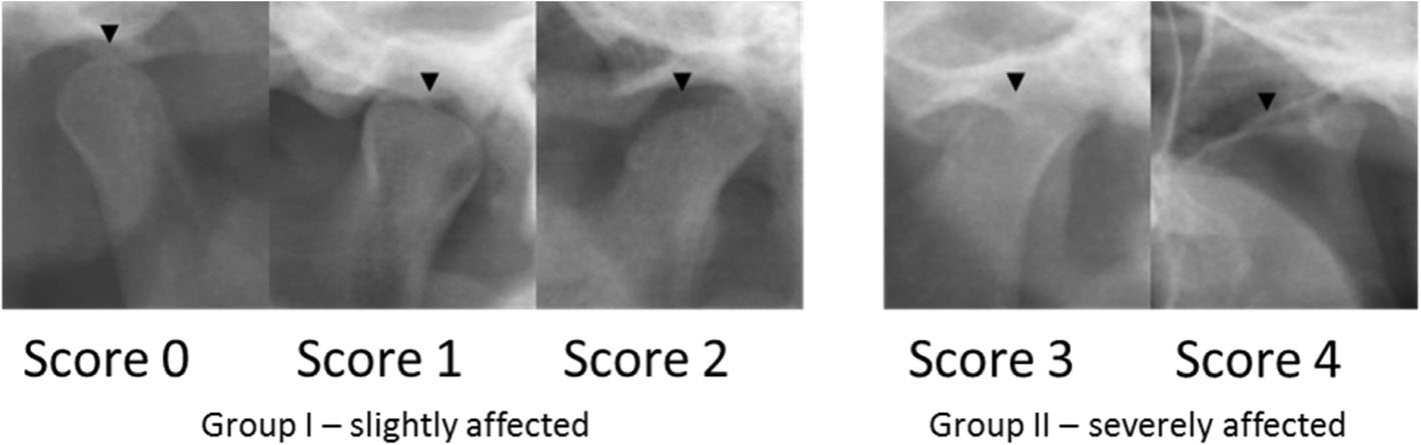
Classification in slightly and severely affected Group according to Billiau et al. [1]. This figure shows the severity of condyle destruction (black arrowhead). Score 0 (radiologically normal appearance; Score 1 (cortical bony erosions); Score 2 (condylar flattening); Score 3 (condylar flattening with additional erosions); Score 4 (complete loss of the condyle). Results with Score 0–2 were classified as slightly affected (Group I), Score 3–4 as severely affected (Group II)

Condyle morphology was analysed according to side (bilateral/unilateral or no involvement) and the severity of condylar destruction (score 0–4). According to Billiau et al., this permits the differentiation of condyles of radiologically normal appearance (score 0) from those with cortical bony erosions (score 1), or flattening (score 2), condylar flattening with additional erosions (score 3) or a complete loss of the condyle (score 4) (Fig. 1) [1]. In the context of the study, results with score 0–2 were classified as slightly affected (Group I) and score 3–4 as severely affected (Group II).

### Asymmetry analysis of the TMJ

**Fig. 2:**
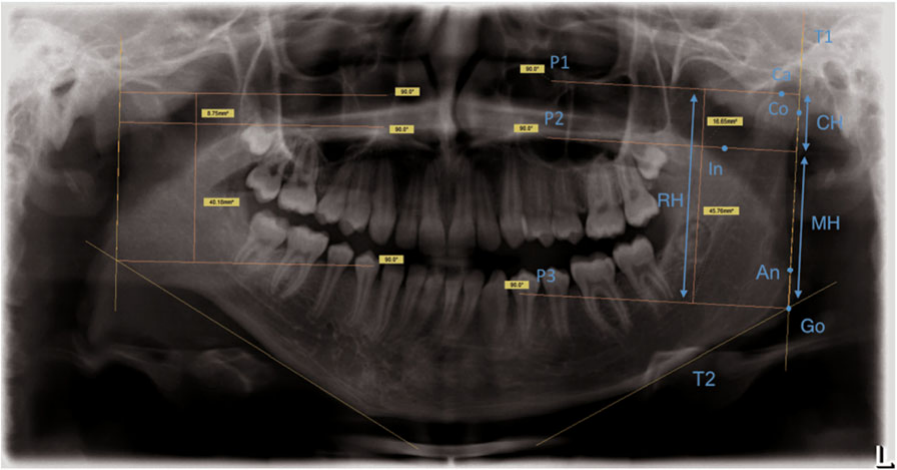
Construction of condyle (CH)-, ramus (RH)- and mandible height (MH) in a pronounced asymmetric mandible with shortened Ramus mandibulae on the right side. Tangent T1 was constructed through the most lateral point of the mandible condyle (Co)- and the angle of the mandible (An). A tangent T2 was drawn on the base of the lower jaw. The intersection of T1 and T2 produced the Gonion (Go). CH, MH and RH could be measured by the constructions of parallels P1, P2 and P3 running vertically to T1 through the highest point of the mandibular head (Ca) mandibular notch (In) and Go

**Fig. 3:**
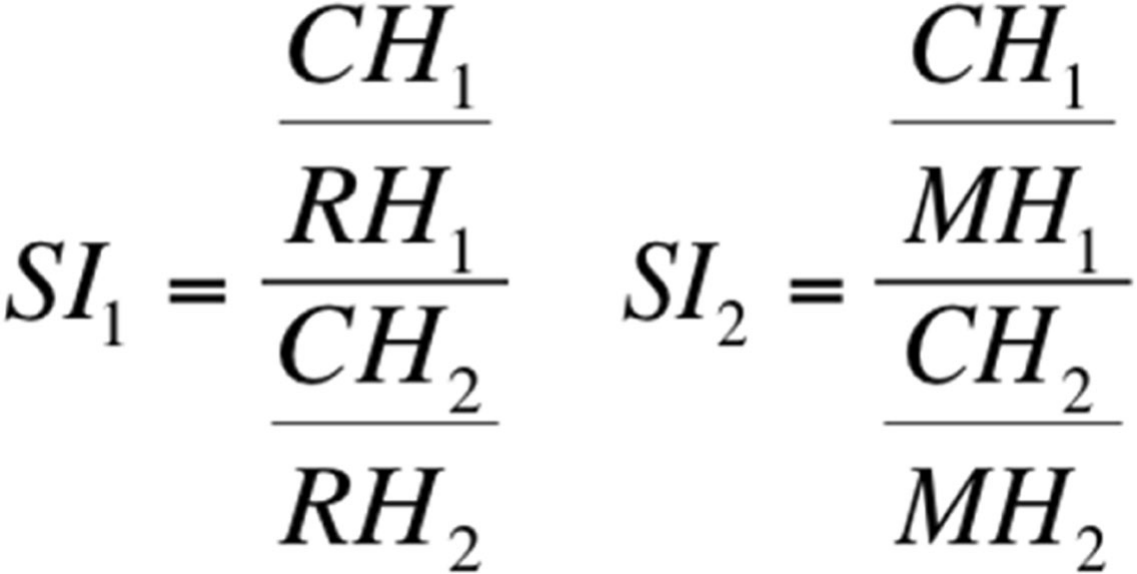
Formulas to form indices S1 and S2 according to Kjellberg et al. [30]

Asymmetry analysis based on bilateral determination of condyle height, ramus height and mandible height, these parameters were used to quantify the asymmetry [30]:

To calculate the asymmetry index, a tangent T1 was first constructed through the most lateral point of the mandibular condyle (Co) and the angle of the mandible (An). A tangent T2 was then drawn on the base of the lower jaw. The intersection of tangents T1 and T2 produced the gonion (Go). Condyle height (CH), mandible height (MH) and ramus height (RH) could then be measured by the construction of parallels P1, P2 and P3 running vertically to T1 through the highest point of the mandibular head (Ca), mandibular notch (In) and gonion (Go) (Fig. 2). The ramus, condyle and mandibular ratio and the degree of lower jaw asymmetry could then be calculated with two indices according to Kjellberg et al. [30]: A ratio of condyle height (CH_1_) to ramus height (RH_1_) of one side of the lower jaw was formed and divided by the ratio of condyle height (CH_2_) and ramus height (RH_2_) of the opposite side of the lower jaw (SI_1_). A second index (SI_2_) was formed in an analogous manner with the ratio of condyle height (CH_1_) to mandible height (MH) (Fig. 3). The indices (SI_1_ und SI_2_) were used to quantify symmetry deviations of the mandibular rami. Reference values for symmetrical ratios have been described with SI_1_ ≥ 93% and/or SI_2_ ≥ 90% [30, 31].

### Statistics

The statistical analysis was performed with the software IBM®, SPSS®, Statistics Version 20.0 for Windows (IBM Deutschland GmbH, Ehningen, Germany). For this clinical explorative case controlled study a normal distribution of the measured primary metric parameters was not provided. Therefore –after testing with the Kolmogorov-Smirnov Test- the non-parametric Mann-Whitney-U-test for independent samples was used to analyse the data for comparing the two groups affected to different extents of severity. A probability of error of *p* < 0.05 was defined as the level of significance.

To determine the error of the method, ten randomly selected OPTs were subjected to repeat cephalometric measurement at an interval of 2 weeks and the error of the method calculated according to Dahlberg [32].

## Results

### Error analysis with the error of the method according to Dahlberg

The error of the method for analysing the cephalometric parameters (condyle height, ramus height and mandible height) was between 0.47–0.97 mm. Overall, this showed that the error of the method was clinically irrelevant for the study and a high reliability of the measurements in the OPT could be achieved.

### Morphological analysis of the condylar destruction

The frequency and distribution pattern of condylar destruction according to Billiau et al. was investigated by morphological analysis (Table 1) [1]. Almost one-third of patients (30%) in the study showed a bilateral involvement of the TMJ, approximately one quarter (22%) showed unilateral involvement and 48% of the patients showed no involvement of the TMJ based on the imaging technique.

**Table 1:** Prevalence of condyle destruction was classified by the Billiau scale [1]. Condyle morphology was analysed according to side. Destruction was classified in two different groups as slightly affected (Group I) and severely affected (Group II)

Prevelance of Condyle destruction according to side	% of patients (*n* = 46)	
Bilateral destruction	30 (14)	
Unilateral destruction	22 (10)	
No involvement	48 (22)	
Prevalence of condyle destruction according to Billiau	% of condyles (*n* = 92)	Classification
Score 0	59 (54)	Group I Slightly affected *n* = 36
Score 1	12 (11)	
Score 2	14 (13)	
Score 3	12 (11)	Gruppe II Severely affected *n* = 10
Score 4	3 (3)	

When the individual condyles were assessed, more than half of the joints (59%) showed a Billiau score of 0 and were therefore recorded as radiologically unremarkable. Eleven joints (12%) were scored 1 and had erosions. Thirteen joints (14%) showed flattening of the condyles and were assigned a score of 2.

**Fig. 4:**
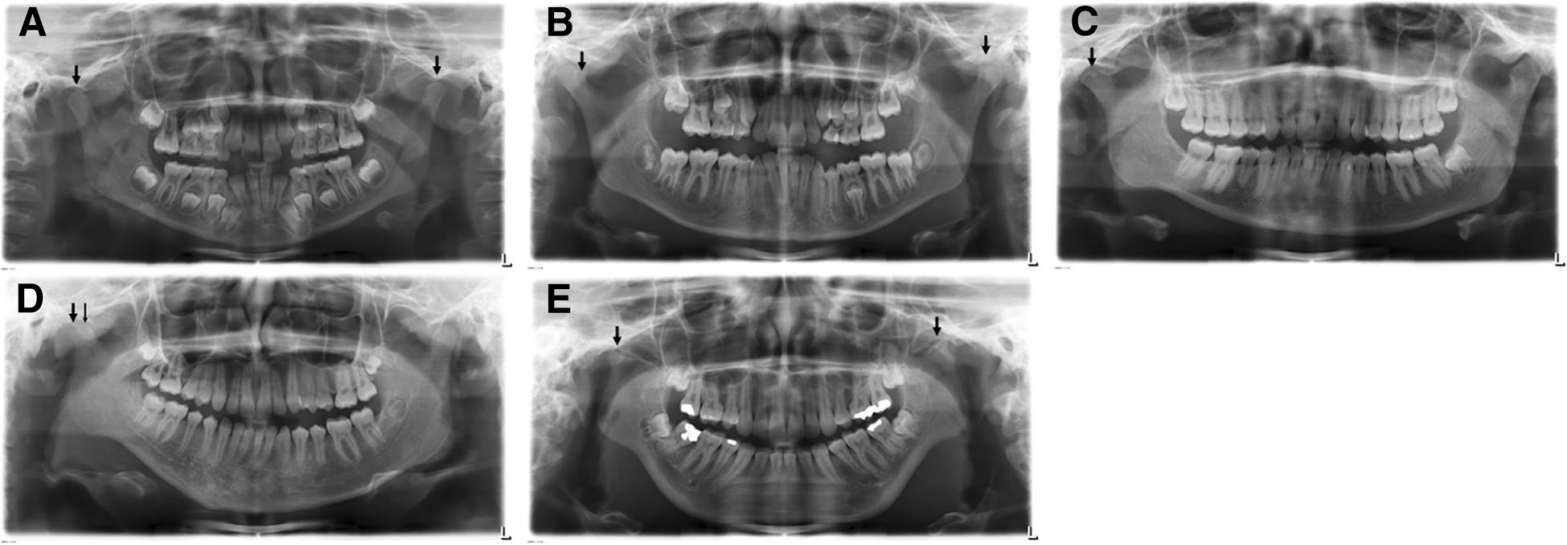
Differing degrees of condylar destruction in patients of this study. **a** Bilateral normal appearance of condyles (Score 0) in a 7 years old girl with RF- polyarthritis and a disease duration of 2,5 years .**b** Bilateral cortical bony erosions (Score 1), more pronounced on the left side. 12 years old girl with an undifferentiated arthritis and a disease duration of 0,2 years. **c** Unilateral condylar flattening (Score 2) on the right side in a 19 years old female patient with psoriatic arthritis and a disease duration of 10,9 years. **d** Unilateral condylar flattening with additional erosions (Score 3) in a 13 years old girl with RF- polyarthritis and a disease duration of 0,2 years. **e** Bilateral complete loss of the condyle (Score 4) in a 16 years old female patient with RF- polyarthritis and a disease duration of 4,7 years

A total of 14 TMJ (15%) were severely affected, of which 11 (12.0%) had a joint destruction score of 3. A score of 4 was assigned to 3 joints (3%). This enabled the patients to be classified into slightly affected (Group I: *n* = 36) or severely affected (Group II: *n* = 10). Figure 4 shows the differing degrees of condylar destruction in patients of this study.

### Disease duration in years

Table 2 shows the chronological age of the patients, subdivided according to condylar destruction into slightly affected (Group I) and severely affected (Group II). There was no significant difference in age between the two groups (*p* = 0.640).

**Table 2:** Disease duration in years. Average, minimal and maximal disease duration in years at 43 of 46 JIA patients with parameter severely and slightly affected condyles. Standard deviation and Median are shown (SD = Standard deviation). The exact time of diagnosis could not be noted for three patients, so the disease duration could not be determied for these patients. Classification was carried out according to Billiau et al. [1]

patients *n* = 43*				Group I *n* = 33 slightly affected	Group II *n* = 10 severely affected	
	Total *n* = 43	Male *n* = 16	Female *n* = 27	Total *n* = 33	Total *n* = 10	*p* slighty vs. severely affected
Age (Years) SD	13.7	13.4	13.9	13.6	14.3	0.640
	3.5	3.8	3.4	3.7	3.0	
Disease duration in years						*p* slighty vs. severely affected
Ø disease duration in years SD Median	5.6	6.1	5.3	4.6	8.9	
	5.1	5.4	5.0	4.7	5.2	0,031
	4.6	4.2	4.6	2.5	9.0	
Minimal disease duration in years	0.2	0.3	0.2	0.2	0.2	
Maximal disease duration in years	17.4	17.4	17.2	17.4	17.2	

*p*-value was determined by Mann-Whitney-U-test

**Fig. 5:**
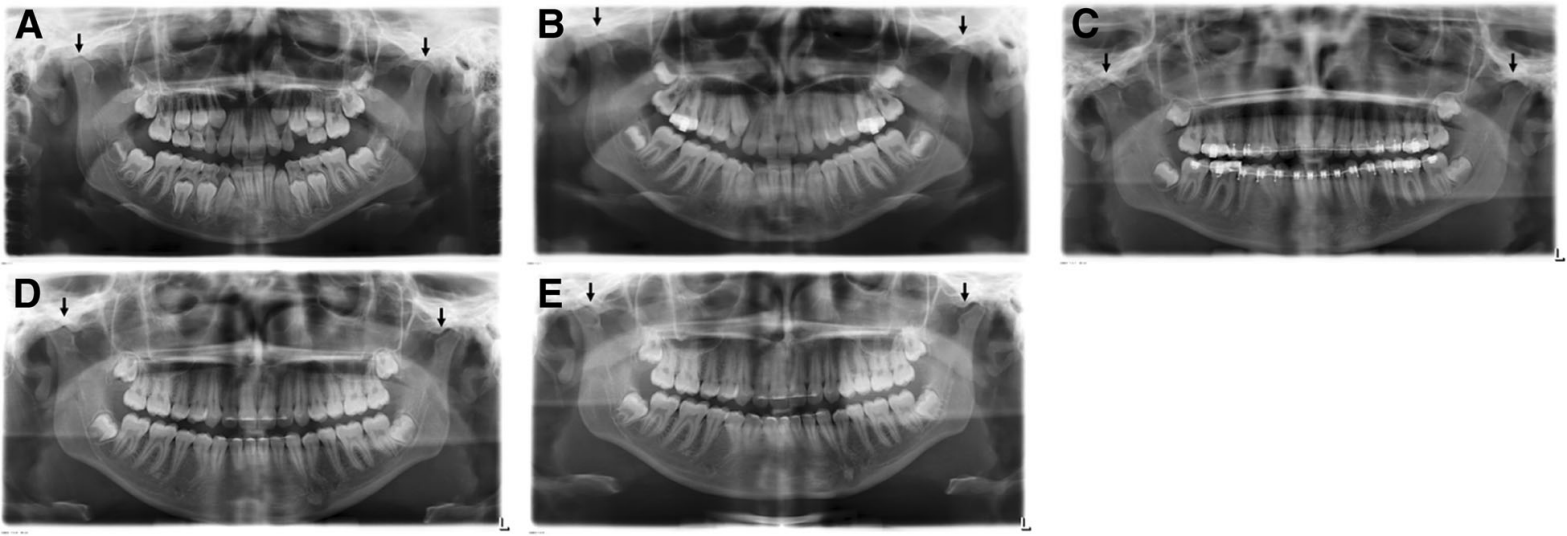
Examples of OPTs of a girl with juvenile, rheumatoid factor negative polyarthritis (initial diagnosis 08/1996) that demonstrate the time course of the disease. The progressive condylar flattening over a period of 8 years is clear. **a** The patient is 10 years old at this time. Condyle morphology appears nearly normal. Only an initial condyle flattening on the left side can be suspected. **b** Two years later there is a condyle flattening on the right side and slightly condyle flattening on the left side. **c** Patient is 14 years old. The left condyle also shows a severe flattening, the right condyle shows a progressive flattening. **d** One year later at the age of 15 the patient shows a clear bilateral flattening. **e** At the age of 18 both condyles are pronounced pathomorphologically and flattened. Bilateral ashortened Processus condylaris can be seen

As no exact date of first diagnosis of JIA was recorded for three patients, their duration of disease could not be determined. The following analysis of the disease duration in years showed an overall mean duration of 5.6 years in all patients (*n* = 43), with a minimum of 0.2 years and a maximum of 17.4 years. There was a significant difference between the average disease duration in the slightly affected Group I (4.6 years (SD ± 4.7 years; range 17.2 years)) and the severely affected Group II (8.9 years (SD ± 5.2 years; range 17.0 years) (*p* = 0.031). Figure 5 shows the example of OPTs of a girl with juvenile, RF- polyarthritis that demonstrate the time course of the disease. The progressive condylar flattening over a period of 8 years is clearly recognizable.

### Asymmetry analysis

Quantitative analysis of the lower jaw asymmetry is shown in Table 3. Considering all patients, the contralateral comparison of condyle, ramus and mandible height and the asymmetry indices produced a reference value of > 90%. Classification of patients according to sex revealed that only females showed reference values of < 90% in the condyle height (ratio 88.9%) and in the asymmetry index SI2 (89.2%).

**Table 3:** Evaluation of OPTs of 46 patients: Quotient of right and left condylus-height in %; quotient of right and left ramus-height in %; quotient of right and left mandible-height in %; Also evalutation of asymmetry indices SI1 and SI2 according to Kjellberg et al. [30]

	patients *n* = 46			Group I *n* = 36 slightly affected	Group II *n* = 10 severely affected	*p* slighty vs. severely affected
	Total *n* = 46	Male *n* = 18	Female *n* = 28	Total *n* = 36	Total *n* = 10	
Age in years	13.6 (3.5)	13.0 (3.8)	14.0 (3.4)	13,4 (3.7)	14.3 (3.0)	0.555
OPT						
Ratio condylus-height in %	90.7 (10.4)	93.6 (6.2)	88.9 (12.2)	93.8 (6.9)	79.6 (13.5)	0.0005
Ratio ramus-height in %	93.8 (6.9)	95.7 (3.7)	92.6 (8.1)	96.0 (4.0)	85.9 (9.3)	0.0030
Ratio mandible-height in %	93.7 (6.6)	95.5 (5.1)	92.4 (7.2)	95.6 (4.0)	86.5 (9.0)	0.0002
Asymmetry index SI1	92.9 (6.6)	94.2 (4.7)	92.1 (7.5)	94.6 (4.4)	87.1 (9.7)	0.0120
Asymmetry index SI2	90.2 (8.4)	91.6 (6.7)	89.2 (9.4)	92.1 (6.0)	83.1 (11.8)	0.0240

p-value was determined by Mann-Whitney-U-test

Significant differences were also found when patients were divided into slightly and severely affected groups. The contralateral comparison of condyle, ramus and mandible height showed markedly lower values and hence greater asymmetries in the severely affected group (79.6–86.5%). The difference in the condyle height was greatest at 14.2%, with a ratio of 93.8% in the slightly affected and a ratio of 79.6% in the severely affected patient group. The asymmetry indices (SI_1_/SI_2_) showed significantly lower values in the severely affected group. With values of 87.1% (SI_1_) and 83.1% (SI_2_), the indices clearly missed the minimum for symmetric relationships of SI_1_ ≥ 93% and/or SI_2_ ≥ 90%. In the slightly affected group of patients, symmetry of the right and left sides of the mandible could be determined, with values of 94.6% (SI_1_) and 92.1% (SI_2_).

## Discussion

In this investigation, condyle pathomorphology of the TMJ in patients with JIA was systematically analysed on the basis of OPTs. Patients were classified according to the destruction score of Billiau, with analysis of the disease duration and the grade of asymmetry of the mandibles as assessed by Kjellberg [1, 30]. Disease duration was defined as period of time since diagnosis of JIA by a pediatric rheumatologist. To keep this in mind it is also one of the limitations of this study: JIA could have existed before the diagnosis. In this case it is not possible to mark a defined beginning of the disease.

Based on the morphological analysis of condylar destruction patients were classified into two groups (Group I – slightly affected, *n* = 36; Group II – severely affected, *N* = 10). The disease duration was significantly longer in Group II (8.9 ± 5.2 years) than in Group I (4.6 ± 4.7 years). Asymmetries of condyle, ramus and mandible height, quantitatively analysed by contralateral comparison, were significantly more marked in patients of Group II than of Group I.

It was possible to analyse bony structures of the TMJ based on the OPT in all patients and classify them into one group with pronounced condyle destruction (scores 3 and 4, severely affected) and another with no/mild destruction (scores 0–2, slightly affected). Thirty percent of all patients showed bilateral involvement of the TMJ and 22% unilateral involvement. These results confirm the data published to date, which show that bilateral involvement of the TMJ is to be expected in a majority of JIA patients [1]. There appears to be an association between the bilateral destruction of the TMJ and the duration of JIA [33].

The value of the OPT as a screening technique is therefore clear. Although radiographic imaging is unable to demonstrate any active inflammatory process [34], the OPT can be useful, firstly during the general dental and orthodontic initial examination and secondly as the first overview of the TMJ. It enables the clinician to assess condyle morphology and, if applicable, to analyse early indicators of a pathological condyle.

One limitation of the present study in diagnostics is, that the destruction score of Billiau could be affected by a subjective bias. Moreover, generally no data exists concerning radiographic guidelines for TMJs of JIA patient. Only a clinical screening protocol for rheumatologists is described, without X-ray diagnostics [35]. Furthermore, there are neither standardized protocols defining the time intervals between routine clinical examinations, nor specific validated examination protocols for JIA patients [14, 25].

But similar to Biliau’s classification Arvidsson et al. graded the TMJ with four grades: normal- small abnormality – moderate- extensive in longitudinal trial up to 27 years in patients with JIA. They found a correlation between an abnormal condylar morphology in the OPG and the occurrence of TMJ synovitis on MRI [36].

According to these recent findings a morphological index such as Billiaus classification could give a first advise directive for radiographic studies of the temporomandibular joint. However, further studies should be carried out in order to examine the validity more closely and to consider further diagnostic criteria.

Magnetic resonance imaging (MRI) has established itself as the diagnostic gold standard for evaluating the TMJ [37, 38]. A close correlation between OPT and MRI findings has already been proved [39]. Abnormal condyle morphology appears to be significantly associated with an increased probability of synovitis in the TMJ [6].

In case of abnormal condyle morphology in the OPT and a confirmed diagnosis of JIA in the anamnesis MRI should be considered subsequently to detect an active inflammatory process.

Fifty percent of the patients in this study showed abnormal condyle morphology in their OPTs. TMJ arthritis cannot be detected on an OPT until it has reached an advanced stage when bony lesions are already present [25], therefore a pathological destructive process of the condyles has to be assumed in these patients.

In addition, comparison of condyle pathomorphology (degree of destruction) with disease duration revealed that patients with marked destruction of the condyles had a significantly longer disease duration than patients with no/mild degree of destruction. This relationship is confirmed by data from the literature. For example, disease duration and disease activity in JIA patients are associated with changes to the TMJ [40]. Bilateral destruction of the TMJ, in particular, is correlated with the duration of JIA [33]. The disease duration not only appears to be associated with the severity of joint involvement, but also with an increased risk of TMJ arthritis [9, 41]. The results of Billiau et al. demonstrate the unpredictable and often insidious character of TMJ arthritis, as well as the risk of progressive condylar destruction. Accordingly, even minimal condylar lesions affect mandibular growth and can lead to craniofacial changes [1].

The results of the present study showed marked asymmetries of the lower jaw. An unequal development was particularly obvious in the severely affected group, with a discrepancy in the vertical dimension of > 30% in the condyle height, > 14% in the ramus and > 13% in the mandible height respectively (Table 3). Further, it would have been useful to examine the Angle’s classes of the patients. JIA patients often have Angle Class II/ 1 malocclusion abnormalities due to mandibular retrognathia, which can also occur in healthy children. Participation of JIA’s temporomandibular joint may therefore go undetected unless patients complain of symptoms [42].

Some authors doubt that exact measurement of condyle and ramus length is possible [43, 44]. Already published data, however, indicate an acceptable accuracy of the OPT for measuring vertical dimensions: vertical asymmetries in the OPT of up to 6% between the right and left side can lead to a deviation of the head position of up to 10 mm. However, a measured discrepancy of more than 6% indicates condylar asymmetries [45].

The Kjellberg asymmetry indices used in our study have proven to be reliable [46]. This is supported by the data presented here with an asymmetry of SI1 = 87% and SI2 = 83%, pathological growth disorders in patients with pronounced condyle destruction are confirmed by the results.

Despite more highly developed, three-dimensional imaging techniques, the OPT still has an important role in the long-term diagnosis of TMJ arthritis in JIA, as shown in a follow-up study [36]. Furthermore, the OPT is being discussed as a screening method in JIA patients [28, 29], which is further supported by our results. The temporomandibular joint involvement in JIA leads to a reduction in the quality of life [21, 22]. The likelihood of a temporomandibular joint infection increases with the duration of the JIA [22]. For this reason, early detection of the disease is particularly important. For clinical aspects the results of our study support the fact that the OPG as a routine recording in the dental and orthodontic treatment can certainly lead to an earlier diagnosis of JIA in the temporomandibular joint. In the long an early diagnosis and the resulting therapy could lead to an improvement in the quality of life of these patients. Further research on this topic should be carried out in the near future.

OPTs can also be an important reference at the start of TMJ pathology for monitoring disease progression during growth [29]. Admittedly, OPTs are unsuitable for the short-term monitoring of joint changes, but this technique is suitable for prevalence studies and as a screening method [34]. Hence this region should be thoroughly investigated at every initial dental or orthodontic analysis in every patient [41].

## Conclusions

In conclusion although MRI is still the gold standard of diagnosis in JIA, OPG is well suited to detect and classify pathomorphological changes in temporomandibular joints in JIA patients and for quantifying asymmetries already in dental and orthodontic routine. In connection with the disease duration and severity of the manifestation of JIA in the TMJ, therapy management could be adjusted at an early stage through this early detection of TMJ infestation.
